# Multi-chaperone function modulation and association with cytoskeletal proteins are key features of the function of AIP in the pituitary gland

**DOI:** 10.18632/oncotarget.24183

**Published:** 2018-01-11

**Authors:** Laura C. Hernández-Ramírez, Rhodri M.L. Morgan, Sayka Barry, Fulvio D’Acquisto, Chrisostomos Prodromou, Márta Korbonits

**Affiliations:** ^1^ Centre for Endocrinology, Barts and The London School of Medicine, Queen Mary University of London, London, EC1M 6BQ, UK; ^2^ Genome Damage and Stability Centre, University of Sussex, Brighton, Falmer, BN1 9RQ, UK; ^3^ Centre for Microvascular Research, Barts and The London School of Medicine, Queen Mary University of London, London, EC1M 6BQ, UK; ^4^ Present address: Section on Endocrinology and Genetics, Eunice Kennedy Shriver National Institute of Child Health and Human Development (NICHD), National Institutes of Health (NIH), Bethesda, MD 20892-1862, USA; ^5^ Present address: Protein Crystallography Facility, Centre for Structural Biology, Flowers Building, Department of Life Sciences, Imperial College London, London, SW7 2AZ, UK

**Keywords:** AIP, co-chaperone, quantitative mass spectrometry, acromegaly, FIPA

## Abstract

Despite the well-recognized role of loss-of-function mutations of the aryl hydrocarbon receptor interacting protein gene (*AIP*) predisposing to pituitary adenomas, the pituitary-specific function of this tumor suppressor remains an enigma. To determine the repertoire of interacting partners for the AIP protein in somatotroph cells, wild-type and variant AIP proteins were used for pull-down/quantitative mass spectrometry experiments against lysates of rat somatotropinoma-derived cells; relevant findings were validated by co-immunoprecipitation and co-localization. Global gene expression was studied in *AIP* mutation positive and negative pituitary adenomas via RNA microarrays. Direct interaction with AIP was confirmed for three known and six novel partner proteins. Novel interactions with HSPA5 and HSPA9, together with known interactions with HSP90AA1, HSP90AB1 and HSPA8, indicate that the function/stability of multiple chaperone client proteins could be perturbed by a deficient AIP co-chaperone function. Interactions with TUBB, TUBB2A, NME1 and SOD1 were also identified. The AIP variants p.R304* and p.R304Q showed impaired interactions with HSPA8, HSP90AB1, NME1 and SOD1; p.R304* also displayed reduced binding to TUBB and TUBB2A, and *AIP*-mutated tumors showed reduced *TUBB2A* expression. Our findings suggest that cytoskeletal organization, cell motility/adhesion, as well as oxidative stress responses, are functions that are likely to be involved in the tumor suppressor activity of AIP.

## INTRODUCTION

The aryl hydrocarbon receptor interacting protein (AIP) is a highly conserved co-chaperone with a poorly characterized tumor suppressor function [1–3]. Germline mutations in the *AIP* gene (*AIP*muts) resulting in a truncated and/or unstable AIP protein are the most common genetic cause of pituitary adenomas affecting teenagers and young adults, presenting either as familial isolated pituitary adenoma (FIPA) or as simplex cases of acromegaly or gigantism [4–7]. Despite the ubiquitous expression of the AIP protein, no other tumor types have been consistently associated with *AIP*muts. This apparent tissue selectivity could perhaps be explained by a tumor suppressor function that is due to pituitary-specific interactions, rather than a tissue-specific expression pattern.

The human AIP protein (UniProt O00170) is a 37 kDa/330 amino acid protein composed of an amino (N)-terminal peptidyl-prolyl cis-trans isomerase (PPIase)-like domain, with no enzymatic activity, and a carboxyl (C)-terminal tetratricopeptide repeat (TPR) domain containing three TPR motifs and a C-terminal alpha 7 helix [8–12]. The best-known function of AIP is to form part, together with the heat-shock protein HSP90 and the co-chaperone translationally-controlled tumor protein (TCTP), of the multiprotein complex that regulates the nuclear translocation of the aryl hydrocarbon receptor (AHR) [8, 13–19]. In addition to HSP90, AIP also serves as a co-chaperone for other molecular chaperones, such as HSPA8 and mitochondrial import receptor subunit TOM20 homolog (TOMM20) [11, 20–22]. It has been proposed that the AIP/HSPA8 complex would bind unfolded mitochondrial pre-proteins; AIP should facilitate the transfer of such pre-proteins to TOMM20, enabling their folding and mitochondrial translocation [20].

Nevertheless, AIP displays a promiscuous repertoire of molecular interactions, including at least two viral proteins, and a variety of human proteins, (Table 1), but none of them has an obvious pituitary-specific function (reviewed in [23]) [24–26]). To elucidate the organ-specific physiological function of AIP in the pituitary gland, and to explore the role of AIP in the pathways that drive pituitary tumorigenesis, we aimed to identify the molecular partners of AIP in the pituitary gland using a proteomic approach.

**Table 1:** Proven and putative human interacting partners of AIP (human proteins only)^*^

UniProt entry	Protein name (gene)	Experimental method	References
P60709	Actin, cytoplasmic 1 (*ACTB*)^¶^	AC-MS	[64]
Q9UL18	Argonaute RISC catalytic component 1 (*AGO1*)	AC-MS, co-IP	[95]
P35869	Aryl hydrocarbon receptor (*AHR*)	Co-IP	[8, 64, 96]
		RC	[8, 9, 16, 97]
		2H	[8, 16, 98]
P27540	Aryl hydrocarbon receptor nuclear translocator (*ARNT*)^†^	Co-IP	[64]
		RC	[16, 57]
		2H	[9]
O15392	Baculoviral IAP repeat-containing protein 5 (*BIRC5*)	AC-MS, co-IP	[21]
Q9BXL7	Caspase recruitment domain-containing protein 11 (*CARD11*)	2H, co-IP	[99]
Q96HB5	Coiled-coil domain containing protein 120 (*CCDC120*)	AC-MS	[100]
Q16543	Hsp90 co-chaperone cell division cycle 37 (*CDC37*)	AC-L, AC-MS	[101]
P50750	Cyclin-dependent kinase 9 (*CDK9*)	AC-MS	[61, 100]
Q9BXN2	C-type lectin domain family 7, member A (*CLEC7A*)	2H	[102]
P68400	Casein kinase 2, alpha 1 polypeptide (*CSNK2A1*)	BA	[103]
Q9NVR5	Protein kintoun (*DNAAF2*)	AC-L, AC-MS	[101]
P00533	Epidermal growth factor receptor (*EGFR*)	PCA	[104]
P41091	Eukaryotic translation initiation factor 2 subunit 3 (*EIF2S3*)	Co-F	[105]
P03372	Oestrogen receptor (*ESR1*)	Co-IP	[106]
Q9UK99	F-box only protein 3 (*FBXO3*)	Co-IP, AC-MS	[7]
P50395	Rab GDP dissociation inhibitor beta (*GDIB*)	Co-F	[107]
Q14344	Guanine nucleotide-binding protein subunit alpha 13 (*GNA13*)	RC, 2H	[108]
P50148	Guanine nucleotide-binding protein G(q) subunit alpha (*GNAQ*)	RC	[108]
P11142	Heat shock cognate 71 kDa protein (*HSPA8*)	Co-IP	[20]
P07900	Heat shock protein 90-alpha (*HSP90AA1*)	AC-L, AC-MS	[101]
		RC	[52, 57, 97]
P08238	Heat shock protein 90-beta (*HSP90AB1*)	AC-L, AC-MS	[101]
		Co-IP	[9, 18, 20, 55, 57, 97, 109–111]§
P38646	Stress-70 protein, mitochondrial (*HSPA9*)	Co-F	[105]
Q9Y6K9	NF-kappa-B essential modulator (*IKBKG*)	2H	[112]
Q92985	Interferon regulatory factor 7 (*IRF7*)	AC-MS, RC	[113]
		Co-IP	[114]
P11279	Lysosome-associated membrane glycoprotein 1 (*LAMP1*)	Co-F	[105]
Q9NZR2	Low-density lipoprotein receptor-related protein 1B (*LRP1B)*	2H, co-IP	[115]
Q9NXB0	Meckel syndrome type 1 protein (*MKS1*)	PL-MS	[116]
Q6IA69	Glutamine-dependent NAD(+) synthetase (*NADSYN1*)	AC-L, AC-MS	[101]
Q86SG6	Serine/threonine-protein kinase Nek8 (*NEK8*)	PL-MS	[116]
Q8WWR8	Sialidase-4 (*NEU4*)	AC-MS	[100]
O75161	Nephrocystin-4 (*NPHP4*)	PL-MS	[116]
P08235	Mineralocorticoid receptor (*NR3C2*)	Co-IP	[111]
O75665	Oral-facial-digital syndrome 1 protein (*OFD1*)	PL-MS	[116]
P27815	cAMP-specific 3’,5’-cyclic phosphodiesterase 4A (*PDE4A*)	2H	[30, 90, 117]
		Co-IP	[117]
O00408	cGMP-dependent 3’,5’-cyclic phosphodiesterase 2A (*PDE2A*)	2H, co-IP, co-loc	[118]
Q15181	Inorganic pyrophosphatase (*PPA1*)	Co-F	[119]
Q9H2U2	Inorganic pyrophosphatase 2, mitochondrial (*PPA2*)	Co-F	[119]
Q07869	Peroxisome proliferator-activated receptor alpha (*PPARA*)	Co-IP, RC	[120]
O75170	Serine/threonine-protein phosphatase 6 regulatory subunit 2 (*PPP6R2*)	PL-MS	[121]
O60809	PRAME family member 10 (*PRAMEF10*)	AC-MS	[100]
Q5VTA0	PRAME family member 10 (*PRAMEF17*)	AC-MS	[100]
P48147	Prolyl endopeptidase (*PREP*)	Co-F	[119]
Q15185	Prostaglandin E synthase 3 (*PTGES3*)	AC-L, AC-MS	[101]
		Co-IP	[111]
P07949	Rearranged during transfection tyrosine-kinase receptor (*RET*)	Co-IP, PCA	[122]
P31948	Stress-induced phosphoprotein 1 (*STIP1*)	RC	[52]
Q9Y2Z0	Suppressor of G2 allele of SKP1 homolog (*SUGT1*)	AC-L, AC-MS	[101]
P13385	Teratocarcinoma-derived growth factor 1 (*TDGF1*)	AC-MS	[100]
Q59H18	TNNI3 interacting kinase (*TNNI3K*)	2H	[123]
Q15388	Mitochondrial import receptor subunit TOM20 homolog (*TOMM20*)	2H, co-IP	[20]
		RC	[11]
P10828	Thyroid hormone receptor beta (*THRB*)	2H	[124]
P0CG48	Polyubiquitin C (*UBC*)	AC-MS	[125–129]
Q6PHR2	Serine/threonine-protein kinase ULK3 (*ULK3*)	AC-MS	[100]
O94966	Ubiquitin specific peptidase 19 (*USP19*)	AC-L, AC-MS	[101]
P07947	Tyrosine-protein kinase Yes (*YES1*)	AC-MS	[100]

Abbreviations: 2H: two-hybrid assay, AC-L: Affinity capture-luminescence, AC-MS: affinity capture (pull-down)-mass spectrometry, BA: biochemical activity (an interaction is inferred from the biochemical effect of one protein upon another), co-F: co-fractionation, co-IP: co-immunoprecipitation (affinity capture-Western blot), co-loc: co-localization, PCA: protein-fragment complementation assay, PL-MS: proximity label-mass spectrometry, RC: reconstituted complex (an interaction is detected between two proteins *in vitro*). Detailed explanation of all the experimental methods: http://wiki.thebiogrid.org/doku.php/experimental_systems.

^*^Includes proteins detected either by direct protein-protein interaction experiments or as part of datasets from high-throughput proteomic studies reported in the literature and/or in the Biological General Repository for Interaction Datasets [26].

^¶^Contradictive results, a direct interaction between AIP and ACTB was disproven by a different study [65].

^†^Contradictive results, a direct interaction between AIP and ARNT was disproven by different studies [8, 16, 109].

^§^These studies did not specify the isoform of HSP90 analyzed or used a peptide that is common to HSP90AA1 and HSP90AB1.

## RESULTS

### Candidate AIP partners and differential interactions among AIP variants

We synthesized N-terminally glutathione-S-transferase (GST)-tagged AIP proteins, including the human wild-type (WT) protein and the variants p.C238Y, p.K266A, p.A299V, p.R304* and p.R304Q (Table 2), and a GST-only negative control. Using these proteins as baits, pull-down experiments were performed in lysates from rat somatotropinoma-derived cells (GH3). Tandem mass tags were used to label the bound proteins, which were pooled together and analyzed by mass spectrometry (MS). Results were filtered for significance, normalized against the negative control, and compared against the WT experiment. Human homologues of these candidate AIP partners were identified and grouped in signaling pathways.

**Table 2:** *AIP* variants selected for the study

Variant^*^	Minor allele frequency (ExAc)^*^	Location in protein	Clinical data	Experimental data	Classification
c.713G>A, p.C238Y (chr11:67257854G>A)	0.000008	TPR2 domain	Detected in one FIPA family with three cases of acromegaly (1.4% of the *AIP*mut positive cases in our cohort) [6, 90, 130, 131].	Results in an unstable protein, probably due to abnormal packaging of the alpha and beta-helices in the second TPR motif [7, 11], with reduced ability for blocking cell proliferation [90], as well as complete disruption the AIP-PDE4A5 interaction [30, 131]. This variant is unable to rescue the lethality caused by *CG1847* knockout in a fruit fly model [132].	Pathogenic
c.796_797delinsGC, p.K266A (chr11:67258267_67258268delinsGC)	NA	TPR3 domain	Not detected in patients.	Disrupts the interaction of AIP with HSP90. Loss of HSP90 binding significantly reduces the ability of AIP to interact with AHR, but it is unknown if this affects other signaling pathways [55].	Experimental
c.896C>T, p.A299V (chr11:67258367C>T)	0.000428	TPR3 domain	Clinical evaluation of multiple carriers does not support a pathogenic role for this rare SNP [131]. It was found in *trans* with a truncating *AIP* mutation in two subjects without pituitary adenomas. Detected in 0.8% of the individuals in our cohort (one patient and four unaffected members of a single FIPA family) [6].	Although *in silico* analysis predicted a possible disruption of protein folding, this variant results in a stable protein, displaying only slight reduction in PDE4A5 binding [7, 30, 131]. Acting similarly to the wild-type protein, this variant is able to rescue the lethality in a *CG1847* knockout fruit fly model [132].	Non-pathogenic
c.910C>T, p.R304* (chr11:67258381C>T)	0.000017	TPR3 domain	Most common *AIP* mutation associated with familial and sporadic pituitary adenomas (35.9% of all the *AIP*mut positive cases in our cohort) [6]. Founder effect in the Northern Irish [133, 134] and Northern Italian [135] populations.	This nonsense mutation is translated into a truncated, unstable protein, resulting in half the normal total AIP content in cells from heterozygous carriers [7, 136]. Causes complete disruption of the PDE4A5 binding and loss of the ability of the mutant AIP to block cell proliferation [30, 90], and impaired ability to suppress cAMP signaling in response to forskolin [2].	Pathogenic
c.911G>A, p.R304Q (chr11:67258382G>A)	0.001458^**^	TPR3 domain	Found for the first time in an apparently sporadic case of Cushing’s disease [137], and subsequently in several young-onset and familial cases, this variant is not rare among *AIP* pituitary adenoma patients (second most common in our cohort, found in 7.9% of the *AIP*mut cases) [6]. Two subjects in ExAc carry this variant in homozygosis.	Relatively conservative, changing a longer side chain, positively charged amino acid, to a slightly shorter, uncharged, hydrophilic one at the C-terminal alpha-7 helix [11]. The protein displays normal half-life [7]. Partially disrupts PDE4A5 binding [30]. Retains the ability to rescue the lethality caused by *CG1847* knockout in the fruit fly [132].	Variant of unknown significance

NA, not available.

^*^All the variants are annotated in the GRCh37/hg19 human genome assembly using the reference transcript NM_003977.3.

^**^Includes two homozygous individuals.

The manually validated qualitative MS results (Supplementary Table 1) accounted for a total of 514 different peptides, matching 154 proteins in addition to AIP itself. After applying selection filters, 30 proteins were identified as candidate partners for WT AIP (Table 3). Pathway analysis identified “Remodeling of the epithelial adherens junction” as the top canonical pathway (Figure 1A), including AIP and 19 of its candidate partners within a single network (Figure 1B). As expected from its previously reported unstable behavior [7], the synthetic p.C238Y AIP protein formed aggregates that precluded its use in our experiments. The repertoire of binding peptides varied among the pull-down experiments for the rest of the AIP variants; quantitative differences in the intensity values of the peptides were interpreted as differential binding of the corresponding proteins for each bait AIP protein (Figure 1C and Supplementary Table 2). Proteins selected for further validation experiments (co-immunoprecipitation [co-IP] and co-localization) included both novel AIP interacting partners and known partners with apparent lost interactions in the pull-down experiments for AIP mutants.

**Table 3:** Candidate AIP partners and peptides identified by quantitative MS and their human homologues

#	*R. norvegicus proteins*				*Human homologues*			
	UniProt entry	Protein description (gene name)	Mass (kDa)	Peptides identified	% of identity	UniProt entry	Protein description (gene name)	Mass (kDa)
1	P60711	ACTB_RAT Actin, cytoplasmic 1(*Actb*)	41.7	VAPEEHPVLLTEAPLNPKANR	100	P60709	ACTB_HUMAN Actin, cytoplasmic 1 (*ACTB*)	41.7
				KDLYANTVLSGGTTMYPGIADR				
2	P29419	ATP5I_RAT ATP synthase subunit e, mitochondrial (*Atp5i*)	8.3	ELAEAEDVSIFK	83	P56385	ATP5I_HUMAN ATP synthase subunit e, mitochondrial (*ATP5I*)	7.9
3	P15999	ATPA_RAT ATP synthase subunit alpha, mitochondrial (*Atp5a1*)	59.8	VGLKAPGIIPR	97	P25705	ATPA_HUMAN ATP synthase subunit alpha, mitochondrial (*ATP5A1*)	59.8
4	P35434	ATPD_RAT ATP synthase subunit delta, mitochondrial (*Atp5d*)	17.6	ANLEKAQSELSGAADEAAR	87	P30049	ATPD_HUMAN ATP synthase subunit delta, mitochondrial (*ATP5D*)	17.5
5	P47727	CBR1_RAT Carbonyl reductase [NADPH] 1 (*Cbr1*)	30.6	EDKILLNACCPGWVR	86	P16152	CBR1_HUMAN Carbonyl reductase [NADPH] 1 (*CBR1*)	30.4
				ELLPIIKPQGR				
				GHEAVKQLQTEGLSPR				
				GVHAKEGWPNSAYGVTKIGVTVLSR				
				KFLGDVVLTAR				
				REDKILLNACCPGWVR				
				SCSPELQQKFR				
6	P08649	CO4_RAT Complement C4 (*C4*)	192	ADLEKLTSLSDR	80	P0C0L4	CO4A_HUMAN Complement C4-A (*C4A*)	193
7	Q497C3	CP013_RAT UPF0585 protein C16orf13 homolog	22.6	MVDMPANNKCLIFR	90	Q96S19	CP013_HUMAN UPF0585 protein C16orf13 (*C16orf13*)	22.6
				NKEPILCVLR				
8	P63255	CRIP1_RAT Cysteine-rich protein 1 (*Crip1*)	8.6	GGAESHTFK	97	P50238	CRIP1_HUMAN Cysteine-rich protein 1 (*CRIP1*)	8.5
9	Q68FR6	EF1G_RAT Elongation factor 1-gamma (*Eef1g*)	50.1	KLDPGSEETQTLVR	98	P26641	EF1G_HUMAN Elongation factor 1-gamma (*EEF1G*)	50.1
				AFKALIAAQYSGAQIR				
				ILGLLDTHLKTR				
				KFPAGKVPAFEGDDGFCVFESNAIAYYVSNEELR				
10	D4ABP9	FBX3_RAT F-box only protein 3 (*Fbxo3*)	55.4	EEDLDAVEAQIGCKLPDDYR	91	Q9UK99	FBX3_HUMAN F-box only protein 3 (*FBXO3*)	54.6
				ITNAKGDVEEVQGPGVVGEFPIISPGR				
11	Q99PF5	FUBP2_RAT Far upstream element-binding protein 2 (*Khsrp*)	74.2	KDAFADAVQR	98	Q92945	FUBP2_HUMAN Far upstream element-binding protein 2 (*KHSRP*)	73.1
12	P48721	GRP75_RAT Stress-70 protein, mitochondrial (*Hspa9*)	73.9	EMAGDNKLLGQFTLIGIPPAPR	98	P38646	GRP75_HUMAN Stress-70 protein, mitochondrial (*HSPA9*)	73.7
				QATKDAGQISGLNVLR				
				MPKVQQTVQDLFGR				
				KDSETGENIR				
				QAVTNPNNTFYATKR				
13	P06761	GRP78_RAT 78 kDa glucose-regulated protein (*Hspa5*)	72.3	KSDIDEIVLVGGSTR	98	P11021	GRP78_HUMAN 78 kDa glucose-regulated protein (*HSPA5*)	72.3
				IINEPTAAAIAYGLDKR				
				NQLTSNPENTVFDAKR				
				AKFEELNMDLFR				
14	P34058	HS90B_RAT Heat shock protein HSP 90-beta (*Hsp90ab1*)	83.3	ELISNASDALDKIR	99	P08238	HS90B_HUMAN Heat shock protein HSP 90-beta (*HSP90AB1*)	83.3
15	P63018	HSP7C_RAT Heat shock cognate 71 kDa protein (*Hspa8*)	70.9	MVNHFIAEFKR	99	P11142	HSP7C_HUMAN Heat shock cognate 71 kDa protein (*HSPA8*)	70.9
				QATKDAGTIAGLNVLR				
				NQVAMNPTNTVFDAKR				
				GTLDPVEKALR				
				LIGDAAKNQVAMNPTNTVFDAKR				
16	Q5FVL7	KTU_RAT Protein kintoun (*Dnaaf2*)	89.3	EWYWGLNKDSLEER	65	Q9NVR5	KTU_HUMAN Protein kintoun (*DNAAF2*)	91.1
17	Q9QX69	LANC1_RAT LanC-like protein 1 (*Lancl1*)	45.2	AFPNPYADYNKSLAENYFDSTGR	91	O43813	LANC1_HUMAN LanC-like protein 1 (*LANCL1*)	45.3
18	Q05982	NDKA_RAT Nucleoside diphosphate kinase A (*Nme1*)	17.2	TFIAIKPDGVQR	95	P15531	NDKA_HUMAN Nucleoside diphosphate kinase A (*NME1*)	17.1
19	P19527	NFL_RAT Neurofilament light polypeptide (*Nefl*)	61.3	KGADEAALAR	97	P07196	NFL_HUMAN Neurofilament light polypeptide (*NEFL*)	61.5
				LAAEDATNEKQALQGER				
				FTVLTESAAKNTDAVR				
				AAKDEVSESR				
				QKHSEPSR				
20	P63324	RS12_RAT 40S ribosomal protein S12 (*Rps12*)	14.5	LGEWVGLCKIDR	99	P25398	RS12_HUMAN 40S ribosomal protein S12 (*RPS12*)	14.5
21	P13471	RS14_RAT 40S ribosomal protein S14 (*Rps14*)	16.3	TKTPGPGAQSALR	99	P62263	RS14_HUMAN 40S ribosomal protein S14 (*RPS14*)	16.3
22	P60868	RS20_RAT 40S ribosomal protein S20 (*Rps20*)	13.4	SLEKVCADLIR	100	P60866	RS20_HUMAN 40S ribosomal protein S20 (*RPS20*)	13.4
23	P05765	RS21_RAT 40S ribosomal protein S21 (*Rps21*)	9.1	LAKADGIVSKNF	95	P63220	RS21_HUMAN 40S ribosomal protein S21 (*RPS21*)	9.1
24	P62859	RS28_RAT 40S ribosomal protein S28 (*Rps28*)	7.8	NVKGPVREGDVLTLLESER	100	P62857	RS28_HUMAN 40S ribosomal protein S28 (*RPS28*)	7.8
25	Q6PEC4	SKP1_RAT S-phase kinase-associated protein 1 (*Skp1*)	18.7	KTFNIKNDFTEEEEAQVR	99	P63208	SKP1_HUMAN S-phase kinase-associated protein 1 (*SKP1*)	18.7
26	P07632	SODC_RAT Superoxide dismutase [Cu-Zn] (*Sod1*)	15.9	GGNEESTKTGNAGSR	83	P00441	SODC_HUMAN Superoxide dismutase [Cu-Zn] (*SOD1*)	15.9
27	P85108	TBB2A_RAT Tubulin beta-2A chain (*Tubb2a*)	49.9	INVYYNEAAGNKYVPR	100	Q13885	TBB2A_HUMAN Tubulin beta-2A chain (*TUBB2A*)	49.9
28	Q6P9T8	TBB4B_RAT Tubulin beta-4B chain (*Tubb4b*)	49.8	INVYYNEATGGKYVPR	99	P68371	TBB4B_HUMAN Tubulin beta-4B chain (*TUBB4B*)	49.8
29	P69897	TBB5_RAT Tubulin beta-5 chain (*Tubb5*)	49.7	ISVYYNEATGGKYVPR	100	P07437	TBB5_HUMAN Tubulin beta chain (*TUBB*)	49.7
30	Q9Z270	VAPA_RAT Vesicle-associated membrane protein-associated protein A (*Vapa*)	27.8	FKGPFTDVVTTNLKLQNPSDR	97	Q9P0L0	VAPA_HUMAN Vesicle-associated membrane protein-associated protein A (*VAPA*)	27.9
				QDGPLPKPHSVSLNDTETR				

**Figure 1:**
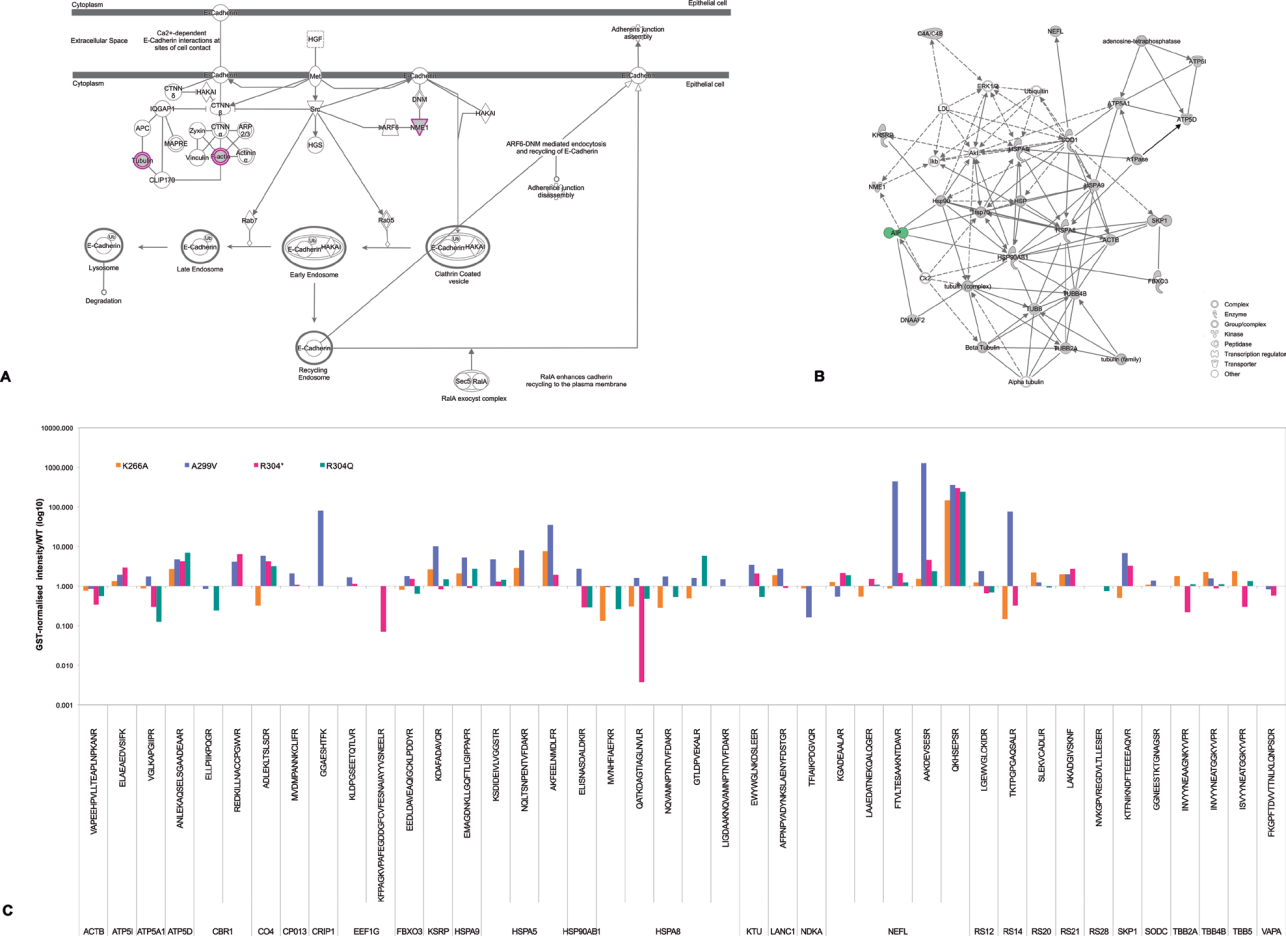
AIP candidate interacting partners: signaling pathways and differential peptide repertoires in pull-down experiments (**A**) The top signaling pathway reported by Ingenuity Pathway Analysis when analyzing the 30 candidate interacting partners identified for WT AIP (as listed in Table 3) was “Remodeling of the epithelial adherens junction” (*P* < 0.0001). This pathway included five candidate partners (ACTB, NME1, TUBB, TUBB2A, and tubulin beta-4B chain [TUBB4B]) out of the total of 68 proteins reported for such pathway by the platform used (overlap: 7.4%). Proteins in this pathway represent multiprotein complexes, with cadherins as central components, mediating cell-cell adhesion and intercellular communication, and regulating cell shape and polarity. Other top canonical pathways reported by this analysis were “Regulation of eIF4 and p70S6K signaling” (5/157 proteins [3.2%], *P* < 0.0001), “EIF2 signaling” (5/194 [2.6%], *P* < 0.0001), “mTOR signaling” (5/199 [2.5%], *P* < 0.0001) and “Protein ubiquitination pathway” (5/255 [2.5%], *P* < 0.0001). Proteins marked in pink are among the AIP candidate interacting partners identified by our pull-down experiments. F-actin refers to “filamentous actin” (i.e. a polymer of actin molecules, including ACTB among other isoforms) which forms part of the cytoskeleton. (**B**) Nineteen of the AIP WT candidate partners were grouped together in a single network, either due to functional relationships or direct binding. (**C**) Schematic representation of the pull-down data presented in Supplementary Table 2 (in a logarithmic scale for easy overview) including only proteins present in the AIP WT experiment. Proteins whose peptides were underrepresented in the AIP variant protein pull-down experiments were interpreted as impaired or lost interactions.

### AIP interacts with multiple molecular chaperones of the HSP70 and HSP90 families

**Figure 2:**
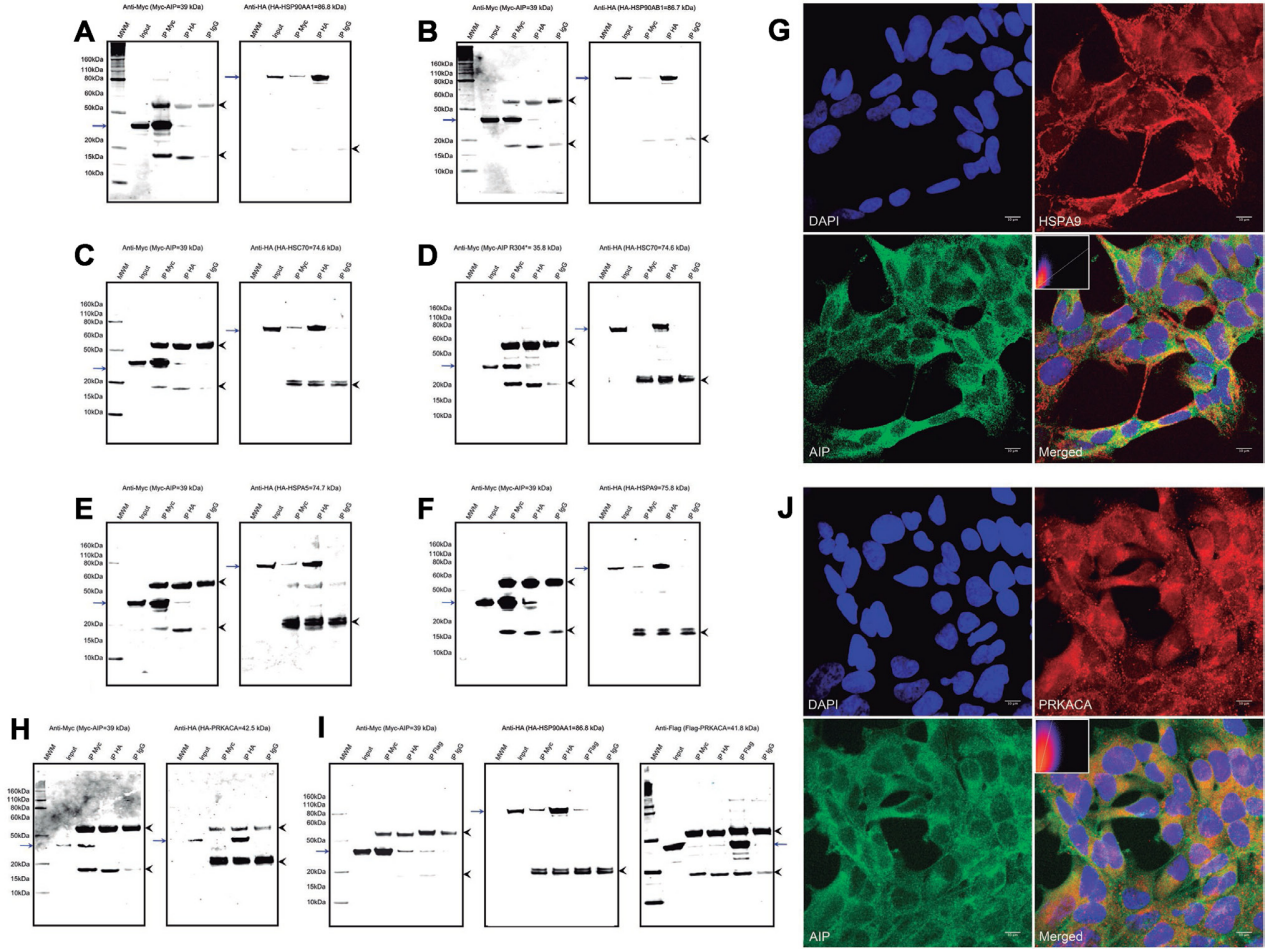
AIP interacts with multiple proteins from the HSP90 and HSP70 families of molecular chaperones WT AIP interactions with HSP90AA1 (**A**) and HSP90AB1 (**B**) were confirmed by co-IP, validating the experimental procedures. Also by co-IP, HSPA8 interacted with WT AIP (**C**), but not with the p.R304* mutant (**D**), as predicted by the pull-down experiment results. Interactions with two novel molecular chaperones were validated by co-IP: HSPA5 (**E**) and HSPA9 (**F**). Co-localization of AIP and HSPA9 (**G**) was confirmed in the mitochondrial network, with a Pearson’s *R*-value of 0.72. (**H**) AIP does not co-immunoprecipitate with PRKACA, ruling out direct interaction of these two proteins. (**I**) However, in the presence of HSP90AB1, the three proteins co-immunoprecipitate with the anti-Myc, anti-HA and anti-Flag antibodies. (**J**) Areas of co-localization for AIP and PRKACA were detected in the cytoplasm, although with a weaker correlation compared with HSPA9, for a Pearson’s *R*-value of 0.39. For the co-IP experiments a-f and h, the left panels represent anti-Myc western blot (WB) membranes and the right panels, anti-HA WB membranes. For the co-IP experiment presented in i the additional panel at the extreme right presents an anti-Flag WB membrane. The blue arrows in all the panels represent the protein of interest in each WB membrane (Myc-AIP on the left panels, and HA-tagged proteins on the right panels, plus Flag-PRKACA in experiment i). The arrowheads in all the panels point out the heavy (top) and light (bottom) chains of mouse immunoglobulins. IP: immunoprecipitation, MWM: molecular weight marker. The immunocytofluorescence images (G and J) are reconstructions of representative images of the z-stacks obtained, at a 63× magnification. Top left: nuclei (DAPI), top right: HSPA9 (G) or PRKACA (J), bottom left: AIP and bottom right: merged image. Inserts in the merged images present 2-D intensity histograms corresponding to the co-localization calculations.

Two of the best-known molecular partners of AIP, the heat-shock proteins HSP90 and HSPA8 (HSC70), were detected in the pull-down experiments. Co-IP experiments for AIP and both the inducible (HSPA90AA1 [HSP90-alpha]) and the constitutive (HSP90AB1 [HSP90-beta]) isoforms of HSP90 [27] and with HSPA8 were carried out and confirmed (Figure 2A–2C). Loss of HSPA8 binding to the AIP mutant p.R304* (Figure 2D) was also confirmed, supporting the reliability of our approach with pull-down experiments and comparative analysis for assuming loss of protein-protein interactions. Novel interactions of AIP with two other members of the HSP70 family, HSPA5 (also known as 78 kDa glucose-regulated protein [GRP-78]) and HSPA9 (mitochondrial HSP70, GRP-75, mortalin), were also detected by pull-down and confirmed by co-IP (Figure 2E and 2F). Co-localization of AIP and HSPA9 in the mitochondrial network was verified by immunocytofluorescence (Figure 2G).

### The pituitary-specific function of AIP could occur via chaperone client proteins

Some pituitary-specific effects of AIP could be exerted via indirect interactions with client proteins of molecular chaperones. Therefore, a possible indirect interaction of AIP with the cyclic adenosine monophosphate (cAMP)-dependent protein kinase catalytic subunit alpha (PRKACA), an HSP90 client protein with an important role in the somatotroph cell function, was explored. AIP and PRKACA did not interact directly (Figure 2H), but when a triple co-IP experiment with AIP, PRKACA and HSP90AA1 was performed, the three proteins were successfully co-immunoprecipitated (Figure 2H–2I), and cytoplasmic co-localization of AIP and PRKACA was observed (Figure 2J).

### AIP is involved in cytoskeletal organization

**Figure 3:**
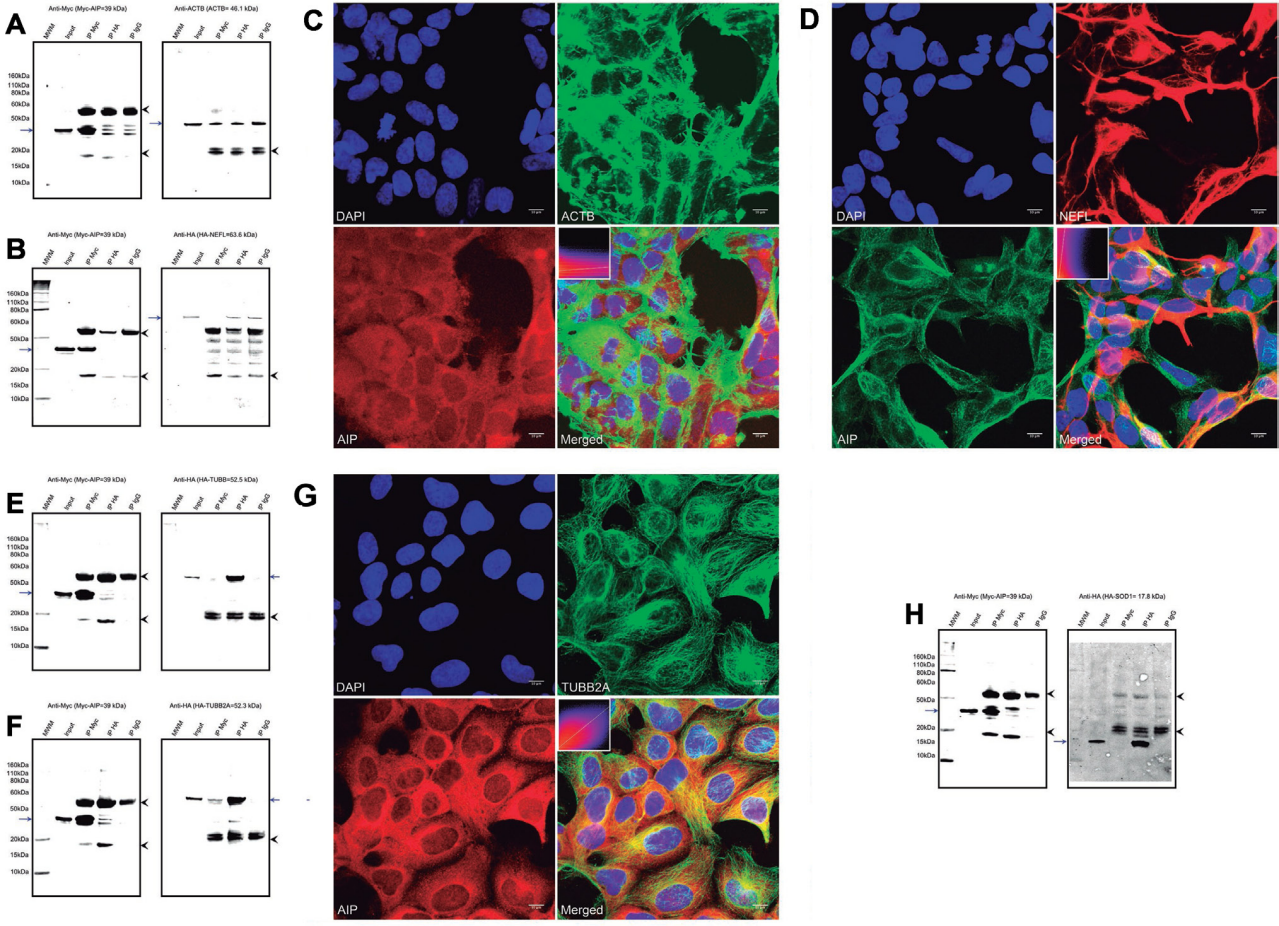
Interactions of AIP with cytoskeletal proteins, the cytoskeletal organizer and tumor suppressor NME1, and the enzyme SOD1 (**A**) A co-IP experiment for AIP and ACTB showed inconclusive results, as the protein was precipitated by the IgG negative control. (**B**) Co-IP for AIP and NEFL rendered negative results. (**C**) ACTB and AIP displayed different distribution patterns in the cell and co-localized only in small perinuclear areas, for a Pearson’s *R*-value of 0.17. (**D**) Likewise, only small areas of co-localization in a few cells were identified for AIP and NEFL, for a Pearson’s *R*-value of 0.38. Positive co-IP experiments were obtained for TUBB (**E**) and TUBB2A (**F**), although the band representing HA-TUBB when immunoprecipitating with an anti-Myc antibody was weak. (**G**) AIP and TUBB2A clearly co-localize in the cytoplasm, particularly in the perinuclear area (Pearson’s *R*-value of 0.54). (**H**) Positive co-IP of AIP and NME1 was observed, although only when the proteins were detected using the anti-Myc antibody. (**I**) Likewise, co-IP of AIP and SOD1 was observed only when detection was performed using the anti-Myc antibody, but not when using the anti-HA antibody. For the co-IP experiments a, b, e, f and h, the left panels represent anti-Myc WB membranes and the right panels, anti-HA WB membranes. The blue arrows in all the panels represent the protein of interest in each WB membrane (Myc-AIP on the left panels, and HA-tagged proteins on the right panels). The arrowheads in all the panels point out the heavy (top) and light (bottom) chains of mouse immunoglobulins. IP: immunoprecipitation, MWM: molecular weight marker. The immunocytofluorescence images (c, d and g) are reconstructions of representative images of the z-stacks obtained, at a 63× magnification. Top left: nuclei (DAPI), top right: ACTB (c), NEFL (d), or TUBB2A (g); bottom left: AIP and bottom right: merged image. Inserts in the merged images present 2-D intensity histograms corresponding to the co-localization calculations.

Multiple cytoskeletal proteins were identified in the pull-down experiments. Although these proteins are abundantly expressed in every cell type and could therefore represent experimental artifacts, some of them displayed differential binding between WT and mutant AIP proteins, suggesting the relevance of these potential interactions. Beta-actin (ACTB) was underrepresented in the pull-down experiments for the AIP variants p.R304Q and p.R304* (normalized intensity values of 0.3 and 0.6, compared with the WT protein). Validation of this interaction was attempted but, although the co-IP reactions were positive in both directions, bands for the co-immunoprecipitated proteins were also observed in the mouse IgG negative control, indicating a non-specific binding of ACTB to this immunoglobulin (Figure 3A). The co-IP experiment between cytoskeletal protein neurofilament light polypeptide (NEFL) and AIP failed to prove the interaction (Figure 3B). In the HEK293 cells, ACTB and AIP displayed different distribution patterns, although some small areas of perinuclear co-localization were observed (Figure 3C), while areas of co-localization for AIP and NEFL were observed in only a few cells (Figure 3D). Interactions of AIP with ACTB and NEFL cannot be discarded, but the experimental artifacts and/or the lack of consistency between co-IP and co-localization complicate data interpretation.

Two isotypes of beta tubulins were under-represented in the pull-down experiment for the mutant p.R304*, compared with WT AIP: tubulin beta chain (TUBB) and tubulin beta-2A chain (TUBB2A, normalized intensity values of 0.2 and 0.3, respectively). Positive co-IP experiments were obtained for TUBB and TUBB2A proteins with AIP, although the band corresponding to TUBB in the anti-Myc immunoprecipitation was weak (Figure 3E–3F). In the confocal immunocytofluorescence images, TUBB2A appeared distributed in cytoplasmic filaments, while AIP had predominantly cytoplasmic diffuse distribution, with clear overlap of both proteins in the cytoplasmic microtubule network (Figure 3G). In addition, a novel interaction between AIP and the cytoskeletal organizer/tumor suppressor NME/NM23 nucleoside diphosphate kinase 1 (NME1) was identified by the pull-down experiment, and binding was reduced for AIP p.A299V (normalized intensity value: 0.2) and lost for the variants p.R304* and p.R304Q. This interaction was validated by co-IP, although only when the anti-Myc antibody was used for protein detection (Figure 3H).

### Interaction of AIP with SOD1

A novel interaction between AIP and the mitochondrial protein superoxide dismutase [Cu-Zn] (SOD1), absent for the variants p.R304* and p.R340Q, was confirmed by co-IP, although a band for SOD1 was not observed in the anti-Myc IP when attempting detection with an anti-HA antibody (Figure 3H), suggesting a weak interaction.

### Negative validation experiments

Negative co-IP experiments were obtained for the candidate AIP partners vesicle-associated membrane protein-associated protein A (VAPA) and carbonyl reductase [NADPH] 1 (CBR1), while inconclusive results (due to immunoprecipitation of the protein of interest with the negative control IgG) were observed for elongation factor 1-gamma (EEF1G). CBR1 binds glutathione and EEF1G contains a GST domain [28]. Therefore, it is likely that these two proteins were pulled down due to the use of a GST/glutathione method and not due to specific AIP binding.

### Differential expression of AIP interacting partners in *AIP*mut positive somatotropinomas

We performed microarray gene expression analysis on samples from *AIP*mut positive and negative human pituitary adenomas and normal pituitaries. We aimed to determine whether the identified AIP interacting partners played a role in pituitary tumorigenesis, independently of *AIP*muts, as well as to determine how the expression profile could be affected by *AIP* deficiency. Comparison of the gene expression profiles of the AIP candidate partners identified in the pull-down experiments revealed that members of the beta tubulin family were differentially expressed. In particular, *TUBB2B* was found significantly downregulated in *AIP*mut positive (−8.3-fold change, *P* = 0.0043) and *AIP*mut negative (−5.2-fold change, *P* = 0.0464) somatotropinomas, compared with the normal pituitaries. Likewise, *TUBB6B* (tubulin beta 6 class V) was significantly downregulated in *AIP*mut negative non-functioning pituitary adenomas (NFPAs, −8.4-fold change, *P* = 0.0057 vs. normal pituitary). By contrast, *TUBB1* (tubulin beta 1 class VI) was upregulated in *AIP*mut positive somatotropinomas (5.16-fold change, *P* = 0.0078 vs. normal pituitary). In addition, upregulation of *NME1* (2.16-fold change, *P* = 0.03) and *HSPA9* (2.37-fold change, *P* = 0.05) was observed in *AIP*mut positive somatotropinomas, compared with normal pituitary. There were no significant differences in these results in *AIP*mut positive compared with *AIP*mut negative pituitary adenomas. Expression of the rest of the AIP partners identified by pull-down was not statistically significantly differentially expressed in *AIP*mut positive, *AIP*mut negative or sporadic somatotropinomas compared with the normal pituitaries. Our full microarray datasets have been deposited at the National Center for Biotechnology Information’s Gene Expression Omnibus (http://www.ncbi.nlm.nih.gov/geo, accession number GSE63357).

### AIP overexpression does not affect AIP interacting partners at the transcriptional level

In contrast to the findings of the microarray analysis, overexpression of WT AIP in HEK293 and GH3 cells (Supplementary Figure 1A and 1B, respectively) displayed no effect on the expression of the interacting partners HSPA5, HSPA8, HSPA9, HSP90AA1, HSPA90AB1, NME1, SOD1, TUBB and TUBB2A at the RNA level at 24 hours post-transfection.

### Overexpressed AIP interacting partners have no effect on GH3 cell proliferation

Individual overexpression of the AIP interacting partners HSPA5, HSPA8, HSPA9, HSP90AA1, HSPA90AB1, NME1, SOD1, TUBB and TUBB2A had no effect on the proliferation of GH3 cells at 24 and 48 h compared with the empty vector control (Supplementary Figure 1C). We also compared the results at the two time points within each experimental condition, finding that overexpression of *TUBB2A* resulted in reduced cell count at 48 h; this effect was not observed for the rest of the genes studied.

## DISCUSSION

We have explored the interactions of AIP with other proteins in pituitary cells, and identified six novel molecular partners for this co-chaperone: HSPA5, HSPA9, TUBB, TUBB2A, NME1 and SOD1. Thus, our results expand the list of possible signaling pathways implicated in the pituitary-specific tumor suppressior function of AIP. Deregulation of the cyclic 3′-5′-adenosine monophosphate/protein kinase A pathway (cAMP pathway) is a well-known initiator of somatotroph cell tumorigenesis [29]. It has recently been suggested that the anti-tumorigenic function of AIP occurs through a regulatory effect on such pathway, mediated by G-inhibitory proteins (Gi_α2_/G_iα3_), very likely coupled to somatostatin receptors (SSTRs) [2, 3], and probably also via phosphodiesterase function regulation [30]. However, while pituitary adenomas/hyperplasia caused by genetic alterations in components of the cAMP pathway (such as *GNAS* in the McCune-Albright syndrome and *PRKAR1A* in Carney complex) share similar molecular, histopathological and clinical characteristics, *AIP*mut-related pituitary adenomas display distinctive features that point to the involvement of additional independent signaling pathways [1, 6, 31].

We have demonstrated interactions between AIP and four different heat shock proteins: two previously known, HSP90 and HSPA8, and two novel molecular partners, HSPA5 and HSPA9. The latter ones have specific subcellular distributions, in sharp contrast with the wide cytoplasmic and nuclear distribution of HSPA8 and HSP90. HSPA5 is a molecular chaperone resident in the endoplasmic reticulum involved in protein quality control and in the unfolded protein response [32]. This cellular response triggers the degradation of unfolded proteins and eliminates cells subjected to prolonged stress but, if sustained, it may elicit the selective proliferation of transformed cells with anti-apoptotic and angiogenic abilities [32–36]. HSPA5 overexpression in cancer is associated with poor prognosis and drug resistance, and represents a novel therapeutic target [37–39]. No previous data exist on the role of HSPA5 on pituitary function, but in a rat gliosarcoma cell line HSPA5 expression is induced by cAMP-responsive protein kinases [40]. If such kinases stimulate HSPA5 expression also in the pituitary gland, this chaperone could amplify the pro-tumorigenic effect of the pathway and the interaction with AIP could regulate its subcellular localization.

Originally identified as a mitochondrial chaperone, HSPA9 is also present in other subcellular localizations, particularly in neoplastic tissues [41–43]. This heat-shock protein is involved in the translocation of proteins from the cytosol into the mitochondrial matrix, in the folding of such imported proteins and in the response to oxidative stress [44, 45]. Deletion of the *HSPA9* locus (5q31.1) is a frequent finding in myeloid malignancies [46]. In other settings, HSPA9 exhibits oncogenic properties, making it a potential therapeutic target for cancer. For instance, overexpressed HSPA9 can sequester TP53 in the cytoplasm [43, 47], inhibit CDKN1A [48, 49], and activate hTERT [50]. Besides the multiple studies addressing the HSPA9 functions in normal and neoplastic cells, little is known about the co-chaperones that regulate its subcellular localization and function, and its expression pattern in the pituitary gland has not been assessed. AIP is a direct partner for TOMM20, located on the mitochondrial surface, and thus the novel interaction with HSPA9 expands the regulatory role of AIP in two different steps of the mitochondrial protein-import machinery [51].

The highly conserved and abundant chaperone HSP90 regulates a wide variety of cellular pathways via interactions with multiple client proteins [52, 53]. When overexpressed in ACTH-secreting pituitary adenomas, HSP90 impairs the sensitivity of the glucocorticoid receptor and therefore adrenal-pituitary negative feedback [54]. However, the role of HSP90 in *AIP*mut-associated pituitary adenomas has not been explored. It has previously been shown that the highly conserved AIP residues G272 and K266 are required for the two proteins to interact [55, 56], but under our experimental conditions, AIP p.K266A did not impair the binding, while mutations affecting the C-terminal alpha-helix of the protein (p.R304* and p.R304Q) did so. Although these variants do not affect the HSP90 binding site directly, an abnormal C-terminal alpha-helix could possibly alter the folding of the third TPR motif, affecting the three-dimensional structure of the TPR-binding site and thus the interaction with HSP90. Previously reported co-IP experiments [57], and now our data, indicate that, despite the fact that the PPIase domain of AIP can also bind HSP90 [58], the integrity of not only the TPR motifs but also of the C-terminal alpha-helix is crucial for this interaction to occur.

Via interactions with multiple co-chaperones, HSPA8 coordinates the dynamics of clathrin-coated vesicles, influences the cell cycle through cyclin D1 and is implicated in protein translocation and quality control [59, 60]. In the absence of the aryl hydrocarbon receptor (AHR), AIP preferentially binds HSPA8 instead of HSP90 and contributes to the prevention of the aggregation of cytosolic proteins, such as mitochondrial pre-proteins synthesized in the cytoplasm, which are then transferred to TOMM20, enabling their folding and mitochondrial translocation [20–22]. HSPA8 was the most consistently represented AIP partner among pull-down experiments, and it was strikingly affected by AIP p.R304*. The physiological importance of this direct interaction between HSPA8 and AIP in the pituitary gland remains undetermined.

The TPR domain of AIP displays binding sites for the MEEVD conserved sequence of HSP90, for the C-terminal motif IEEVD of HSPA8, and EDDVE of TOMM20 [11]. Interestingly, neither HSPA5, nor HSPA9 contain such conserved motifs, and none of the AIP variants studied disturbed these interactions. Given the prominent role of these chaperones in protein folding, a role for AIP as a mediator of the protein quality control system in the pituitary gland could be expected. Both HSP90 and HSPA8 are ubiquitously expressed, but their interacting partners include proteins with tissue-specific functions and/or expression patterns. The interaction between HSP90 and PRKACA was reported previously as part of a high throughput proteomic study, but it was not further functionally or structurally characterized [61]. Our data here support this interaction. The HSP90/AIP complex could feasibly regulate PKA localization, and it could possibly also interact with the regulatory subunits of PKA, as it has recently been proposed [62].

The second group of AIP partners identified is composed of cytoskeletal proteins. While very weak or negative binding of the PPIase-like domain of AIP to dynein was found previously [63], a different study demonstrated that the AIP-mediated cytoplasmic localization of the AHR requires the anchoring of the complex to actin filaments [64]. The latter study showed direct binding of AIP to ACTB when AHR is inactive, but a different group reported conflicting results regarding such an interaction [65]. Unfortunately, we cannot shed light on this issue, due to non-specific binding of ACTB to IgG, although perinuclear co-localization was seen on immunostaining. Likewise, the interaction of AIP with NEFL could not be clearly validated or ruled out (negative co-IP, despite positive co-localization in some cells).

Composed of heterodimers of various alpha and beta-tubulins, microtubules are structures involved in cell movement, intracellular transport (including vesicle trafficking) and cell division and are targets of anti-mitotic drugs [66–69]. In the pituitary gland, the cytoskeletal network plays a crucial role in regulating the function of signal transduction. It has been suggested that low levels of filamin A, a cytoskeletal-associated protein, could represent a post-receptor mechanism of pharmacological resistance in pituitary adenomas, as this scaffolding protein stabilizes the SSTR2 and dopamine 2 receptor, linking them to their intracellular effectors [69, 70]. We validated direct interactions of AIP with two isotypes of tubulins: TUBB, widely expressed, and TUBB2A, the major isotype in neural tissue [67]. Interestingly, two isotypes of beta tubulin were also deregulated (*TUBB1* with five-fold upregulation and *TUBB2B* with eight-fold downregulation) in *AIP*-mutated somatotroph tumors, compared with normal pituitaries. These results point towards a role for AIP as a regulator of the microtubule network, with possible implications for post-receptor signal transduction and hormone secretion.

NME1 (granzyme A-activated DNase, metastasis inhibition factor nm23), the first metastasis suppressor gene identified [71–73], is a tumor suppressor that negatively regulates cell migration/motility and inhibits the cell cycle through downregulation of cyclin B, an effect inhibited by cAMP [74]. This protein phosphorylates kinase suppressor of Ras 1 (KSR1), thereby inactivating the RAS/RAF/MEK/ERK signaling pathway [75]. *NME1* mRNA levels are high in cell lines with low metastatic potential [71], while LOH involving the *NME1* locus (17q21.3) has the opposite effect [76]. Low *NME1* expression correlates with metastasis and poor clinical prognosis in different human epithelial cancer types [77]. Moreover, *NME1* knockdown in various human cancer cell lines disrupts E-cadherin-mediated cell adhesion, leading to nuclear translocation of beta-catenin [78], while its overexpression inhibits the metastatic potential of TP53-deficient cells [73]. NME1 co-localizes with E-cadherin in epithelial cancer cell lines, suggesting a possible role on the stabilization of the adherens junctions [78]. In pituitary adenomas, *NME1* expression inversely correlates with tumor extension into the cavernous sinus [79], but there are no data available on its expression pattern in different pituitary adenoma subtypes. Since *AIP*mut positive pituitary adenomas are frequently invasive, the interaction with AIP should have a regulatory effect on this kinase [80].

Interestingly, “Remodeling of the epithelial adherens junction” was identified as the top molecular pathway encompassing our candidate AIP interacting partners. Loss of the integrity of epithelial adherens junctions results in epithelial-mesenchymal transition (EMT), a process by which polarized epithelial cells develop increased migratory capacity, invasiveness, resistance to apoptosis and increased production of extracellular matrix, ultimately acquiring a mesenchymal cell phenotype [81]. Loss of expression of E-cadherin, the main constituent of the adherens junctions, is a hallmark of EMT. In somatotropinomas, E-cadherin expression correlates positively with GH and IGF-1 secretion and and with somatostatin analogue treatment, and negatively with tumor size and invasiveness [82]. Interestingly, the characteristics of somatotropinomas with low E-cadherin expression recapitulate the phenotype described in the *AIP*mut positive setting (unpublished data from our group). A function of AIP as a regulator of the adherens junctions in the somatotroph cells, suggested by our results, would explain why somatotroph cells acquire an EMT phenotype in the setting of *AIP* deficiency.

Finally, we found that AIP is able to bind the enzyme SOD1, required for the conversion of superoxide to hydrogen peroxide, a mechanism that counteracts oxidative stress [83]. Rat somatotropinoma-derived GH3 cells secrete SOD1, which, via activation of a muscarinic M1 receptor, reduces the activity of the MAPK1 signaling pathway, by inhibiting MAPK3 phosphorylation, reducing cell proliferation [84, 85]. On the other hand, accumulation of free radicals as a result of defective enzyme function can lead to neurotoxicity, as it happens in *SOD1* mutation associated familial amyotrophic lateral sclerosis, or to malignant transformation in different tissues [83]. Molecular chaperones such as HSP70 and HSPA8 regulate SOD1 function and prevent its aggregation [86]. We hypothesize that AIP could form part of this complex, therefore regulating the activity of SOD1.

It was not completely unexpected to find that AIP overexpression has no effect on the expression of its interacting partners at the transcriptional level. Besides the fact that AIP does not have a known direct transcriptional effect, this co-chaperone is highly expressed in the somatotroph cells under basal conditions. Therefore, it is unlikely that AIP overexpression should have any acute transcriptional effects, even if indirect. However, the role of AIP overexpression under certain stimuli, for instance, in somatotropinomas treated with somatostatin analogues, might have a role on the posttranscriptional regulation of other proteins. Likewise, overexpression of multiple AIP interacting partners had no effect on the proliferation of GH3 cells. Overexpression of *TUBB2A* had a small, but significant effect on reducing cell proliferation at 48h compared with the same condition at 24 h. HSPA5, HSPA8, HSPA9, HSP90AA1, HSPA90AB1, NME1, SOD1, TUBB and TUBB2A are highly expressed in the cells and have no known direct oncogenic effects.

We acknowledge the shortcomings of our study. Ideally, the pull-down experiments should had been done in lysates from human pituitary cells, but the lack of commercially available human pituitary cell lines and the difficulty to obtain fresh normal pituitary tissue from autopsy specimens in a quantity enough as to optimize and perform the experiments precluded the use of such material. Although a rat pituitary cell line was used for the pull-down experiments, we overcame this disadvantage by validating our results using human proteins. Finally, concerning the validation experiments, it would be desirable, but extremely impractical, to validate all the protein interactions identified. Interactions of AIP with novel partners have been fully validated, but it is possible that some of the other proteins identified (and not validated) could represent true interactions, providing opportunity for further experiments. While the finding of new chaperones interacting with AIP has shed light on the repertoire of interacting partners of AIP, it has also increased the number of chaperone-client proteins that could be regulated by the co-chaperone activity of AIP. Although mapping all these possible indirect interactions would be complicated, future studies could concentrate on determining which interactions of these chaperones in the somatotroph cells are mediated by AIP.

## CONCLUSIONS

Several novel protein partners were identified and validated for AIP in somatotroph cells. Interactions with HSP70 family members HSPA5 and HSPA9 expand the repertoire of heat-shock proteins that could be modulated by AIP, opening a new window for possible anti-tumorigenic functions of AIP as a regulator of stress-induced heat-shock protein functions. AIP also binds SOD1, an anti-oxidative protein with anti-proliferative potential. In addition, novel molecular interactions with the cytoskeletal proteins TUBB and TUBB2A and the cytoskeletal regulator NME1 indicate a possible role for AIP as a regulator of cytoskeletal organization and on the integrity of the adherens junction, which might be a novel mechanism for the complex tumor suppressor function of AIP in the pituitary gland. The study of this mechanism could be the focus of future studies.

## METHODS

### Protein synthesis and pull-down assays

The human *AIP* variants listed in Table 1 were obtained by site-directed mutagenesis (QuikChange II XL kit, Agilent Technologies, Santa Clara, CA, USA, 200521) from the p-THREE-E-AIP_WT plasmid [7]. Under IPTG induction, WT and mutant GST-AIP proteins were produced in BL21-PLyss *E. coli* and subjected to affinity (Glutathione Sepharose 4 Fast Flow, GE Healthcare, Little Chalfont, UK, 17-5132-02) and gel filtration chromatography. GST was obtained likewise and used as a negative control. Ten micrograms of each synthetic protein were used as baits for individual GST pull-down experiments against 2 mg of total protein from GH3 cells (ECACC, Porton Down, UK, 87012603), and eluates from four independent experiments for each bait protein were pooled together for MS analysis, as reported before [7].

### Quantitative MS and identification of candidate AIP partners

Tandem mass tagging (TMTsixplex Label Reagent Set, Thermo Fisher Scientific, Waltham, MA, USA, 90061) [87], and ion trap tandem MS (Orbitrap Velos Pro, Thermo Fisher Scientific) of the pull-down eluates were carried out at the King’s College London Denmark Hill Proteomics Facility, according to their standard procedures. Data were processed using the Proteome Discoverer version 1.3.0.339 (Thermo Fisher Scientific) software, and the sequences identified were searched in the UniProt database [12] via the Mascot v.2.2 platform [88]. Results were qualitatively analyzed and the MS fragmentation spectra were manually validated using the Scaffold 3.6 (Proteome Software) software. Only peptides with Mascot score ≥20 (to filter out non-significant results) and valid MS spectrum were considered for further analysis. The intensity value of each peptide in the GST experiment was subtracted from the value obtained for the same peptide in the rest of the experiments, and the normalized value for each of the mutants was divided by the normalized value for the WT experiment. Differential binding results are presented as fold-change, considering the results for the WT experiment in each case as 1. Candidate partners were selected based on the Mascot score, the number of unique peptides identified per protein (≥3 peptides was interpreted as highly probable candidates) and the differential intensity values among experiments. The human homologues of the candidate peptides selected were identified using the Blast tool in the UniProt database; the best match was selected in each case. The Ingenuity Pathways Analysis platform [89] was used for grouping the identified proteins in signaling pathways. Results for proteins involved in the ubiquitin-proteasome pathway have been reported elsewhere [7].

### Validation of protein-protein interactions

Plasmids containing the coding sequence for *HSC70, HSP90AA1* and *HSP90AB1* were a kind gift from Prof. Paul Chapple (Barts and The London School of Medicine). The coding sequence for *SOD1* was cloned from human cDNA from HEK293 (human embryo kidney, ECACC 85120602) cells. For the rest of the candidate partners, plasmids were obtained from a repository (PlasmID DNA Resource Core, Harvard Medical School). All the sequences were sub-cloned into the pSF-CMV-NH2-HA-EKT-NcoI plasmid (Oxford Genetics, Oxford, UK, OG93) to express N-terminally HA-tagged proteins. The plasmids pcDNA3.0-Myc-WT_AIP and pcDNA3.0-Myc-AIP-R304* were used to express N-terminally Myc-tagged WT and p.R304* AIP, respectively [90], and the plasmid pSF-CMV-NH2-HA-EKT-Nco1-PRKACA was subjected to site-directed mutagenesis to obtain an N-terminal Flag tag. The final constructs are detailed in Supplementary Table 3.

Each of the plasmids was co-transfected with pcDNA3.0-Myc-WT_AIP plasmid in 10 × 10^6^ HEK293 cells for a total of 20 μg of plasmid DNA, with 1 μl of Lipofectamine 2000 (Invitrogen, Carlsbad, CA, USA, 11668027) per μg of DNA, according to the manufacturer’s instructions. Cells were harvested one day later by trypsinization and resuspended in 1.5 ml of lysis buffer, composed of 150 mM NaCl, 10 mM Tris-Cl pH 7.5, 10% v/v glycerol, 1% v/v IGEPAL CA-630 (Sigma-Aldrich, St. Louis, MO, USA, I8896) and 1 tablet per 50 ml Complete Protease Inhibitor Cocktail (Roche, Basel, Switzerland, 11836145001). After cleared by centrifugation, lysates were cleaned up by incubation with 50 μl of Protein G Sepharose 4 Fast Flow (GE Healthcare 17-0618-01), and divided in thirds for incubation with 5 μg of anti-Myc (Sigma-Aldrich M4439), or anti-HA mouse (Sigma-Aldrich H3663) monoclonal antibodies or mouse anti-IgG (Sigma-Aldrich I5381), as appropriate. Co-IP was performed as previously described [7], and the eluates were resolved by denaturing polyacrylamide gel electrophoresis and mouse anti-Myc and rabbit anti-HA (Sigma-Aldrich H6908) Western blot. A mouse monoclonal anti-ACTB antibody (Sigma-Aldrich A1978) was used for ACTB immunoprecipitation. Detection was performed in an Odyssey Infrared Imaging System (LI-COR, Lincoln, NE, USA) after incubation with secondary infrared fluorescent antibodies (LI-COR 926-68180 and 926-32211). All the co-IP experiments were performed at least twice for confirmation.

### Co-localization

For co-localization experiments, 5 × 10^4^ HEK293 cells per well were plated on an 8-well chamber slide (Thermo Fisher Scientific 154534) and grown for 48 h. Cells were pre-fixed for 2 min with 4% formaldehyde in PBS added to the medium, fixed for 10 more minutes after medium removal, washed thrice with PBS, permeabilized for 20 min at room temperature (0.1% triton X-100 in PBS), blocked for 1 h (10% normal goat serum [VECTOR Laboratories, Burlingame, CA, USA, S-1000] in permeabilization buffer) and incubated overnight with primary antibodies against AIP (mouse monoclonal 1:500 v/v [Novus, Littleton CO, USA, NB100-127], or rabbit polyclonal 1:100 v/v [NBP1-31347], as appropriate) and one of the following: rabbit polyclonal anti-PRKACA (Proteintech, Rosemont, IL, USA, 5388-1-AP, 1:50 v/v), rabbit monoclonal anti-GRP75 (Cell Signalling Technology, Danvers, MA, USA, 3593, 1:50 v/v), rabbit monoclonal anti-NEFL (Cell Signalling Technology 2837, 1:100 v/v) or mouse monoclonal anti-TUBB2A (Abnova, Taipei, Taiwan, H00007280-M03, 1:100 v/v). For actin staining, ActinGreen488 anti-ACTB probe (Invitrogen R37110) was used, following the manufacturer’s protocol, after incubation with the primary anti-AIP antibody. The cells were washed, incubated with 1:500 v/v fluorescent secondary antibodies (Alexa Fluor 488 Goat Anti-Mouse IgG [H+L] green and Alexa Fluor 568 Goat Anti-Rabbit IgG [H+L] orange, Invitrogen A-11029 and A-11036, respectively), washed again, and mounted with 4’,6-diamidino-2-phenylindole-containing mounting medium (UltraCruz Hard-set Mounting Medium, Santa Cruz Biotechnology, Dallas, TX, USA, sc-359850) for confocal microscopy analysis in an LSM 510 (Mark 4) Laser Scanning Confocal Microscope (Zeiss, Oberkochen, Germany). Z-stack images were obtained and visualized using the ImageJ version 2.0.0-rc-54/1.51g software [91]. Co-localization was analyzed on representative images with the Coloc 2 plugin, using the Costes threshold regression method and using the Pearson’s correlation coefficient to quantify co-localization [92–94].

### Microarray expression analysis

Total RNA was extracted from five normal pituitaries (obtained from autopsies) as well as from twenty freshly frozen pituitary adenoma samples, including six somatotropinomas from patients carrying germline *AIP* variants (c.910C>T, p.R304* in three cases, and c.911G>A, p.R340Q, c.100–1025_279_357del, p.A34_K93del and c.100–18C>T in one patient each), seven *AIP*mut negative somatotropinomas and seven clinically NFPAs, using the RNeasy plus mini kit (Qiagen, Hilden, Germany 74134). Patients were recruited as part of our cohort of pituitary adenoma patients and provided signed informed consent [6]. Global gene expression analysis was performed using Human Gene Chip HG-U133 Plus 2.0 arrays (Affymetrix, Santa Clara, CA, USA). A double cut-off of false discovery rate <0.05 and fold change of ≥2 was used to identify differentially expressed genes. The Ingenuity Pathway Analysis platform was used to analyze differentially expressed genes, and results were validated by RT-qPCR. Results were analyzed separately for each subgroup of samples and gene expression profiles were compared to seek differences between *AIP*mut positive and negative somatotropinomas, between each tumor type and the normal pituitary or between functioning tumors (somatotropinomas) and NFPAs. Data are presented only for genes with significant differential expression corresponding to protein families that were present also in the pull-down assays.

### Expression of AIP interacting partners under AIP overexpression

For AIP overexpression experiments, 2.5 × 10^5^ HEK 293 or GH3 cells per well were plated in 12-well plates and transfected 24h later with 1μg of either pcDNA3.1(−) (empty vector), pcDNA3.0-Myc-WT_AIP, or an equivalent volume of water, and 2 μl of TurboFect (Thermo Fisher Scientific R0531) per well, according to the manufacturer’s instructions, in triplicate; each experiment was repeated at least twice for confirmation. RNA was extracted 24 hours later, as described before, and 500 ng of total RNA were reverse-transcribed using the SuperScriptIII First-Strand Synthesis Super Mix (Thermo Fisher Scientific 11752250), following the manufacturer’s protocol. Quantitative PCR was performed in a final volume of 10 μl, with 5 ng of cDNA per reaction, in triplicate, using the TaqMan Fast Advanced Master Mix (Thermo Fisher Scientific 4444557). The FAM-MGB labelled TaqMan expression assays Rn00565250_m1 (*Hspa5*), Rn00821191_g1 (*Hspa8*), Rn01402372_g1 (*Hspa9*), Rn00822023_g1 (*Hsp90aa1*), Rn01511686_g1 (*Hsp90ab1*), Rn00821755_g1 (*Nme1*), Rn00566938_m1 (*Sod1*), Rn00597407_m1 (Tubb5), and Rn01774446_m1 (Tubb2a), were used for experiments in GH cells, and Hs99999174_m1 (*HSPA5*), Hs03044880_gH (*HSPA8*), Hs00945584_m1 (*HSPA9*), Hs00743767_sH *HSP90AA1*), Hs00607336_gH (*HSP90AB1*), Hs00533490_m1 (*SOD1*), Hs02621161_s1 (*NME1*), Hs00742828_s1 (*TUBB*), Hs00742533_s1 (*TUBB2A*), were used for HEK293 cells; Hs066610222_m1 (*AIP*) was used for both sets of experiments. The VIC-TAMRA labelled endogenous controls Rn00667869_m1 (*Actb*) and 4325788 (*ACTB*) were used for GH3 a and HEK293 cells, respectively. Reactions were carried out using the ViiA 7 Real-Time PCR System (Thermo Fisher Scientific) and results were analyzed via the 2^-DDCT^ method, normalized against empty vector control, and compared among experimental conditions.

### Cell proliferation assays under overexpression of AIP interacting partners

For cell proliferation experiments, 2.5 × 10^4^ GH3 cells per well were plated in 96-well plates and transfected 24 h later with 200 ng of HA-tagged plasmids to overexpress AIP interacting partners (Supplementary Table 3), empty vector or water, and 4 μl of Turbo Fect per well, in triplicate. Cell proliferation assays were performed at 24 and 48 h after transfection, using the CyQUANT Direct Cell Proliferation Assay (Thermo Fisher Scientific C35011), according to the manufacturer’s instructions. Fluorescence was detected using a FLUOstar Omega plate reader (BMG Labtech, Offenburg, Germany), normalized against blanks (cell culture medium only) and then against empty vector control, and compared among experimental conditions. Experiments were performed at least twice, for confirmation.

## SUPPLEMENTARY MATERIALS FIGURES AND TABLES









## Abbreviations

AIP: aryl hydrocarbon receptor interacting protein
*AIP* mutation: *AIP*mut
C: carboxyl
cAMP: cyclic 3′-5′-adenosine monophosphate
co-IP: co-immunoprecipitation
FIPA: familial isolated pituitary adenoma
GST: glutathione-S-transferase
IP: immunoprecipitation
MS: mass spectrometry
MWM: molecular weight marker
N: amino
NFPAs: non-functioning pituitary adenomas
PPIase: peptidyl-prolyl cis-trans isomerase
SSTRs: somatostatin receptors
TPR: tetratricopeptide
WB: western blot
WT: wild-type.
