# Supplementary material for: Multi-chaperone function modulation and association with cytoskeletal proteins are key features of the function of AIP in the pituitary gland
Source: Oncotarget. 2018 Jan 11;9(10):9177–98. doi: 10.18632/oncotarget.24183 (PMC5823669; doi:10.18632/oncotarget.24183)
Supplement: Supplementary file 1 [file oncotarget-09-9177-s001.pdf]

Multi-chaperone function modulation and association with cytoskeletal proteins are key features of the function of AIP in the pituitary gland

SUPPLEMENTARY MATERIALS

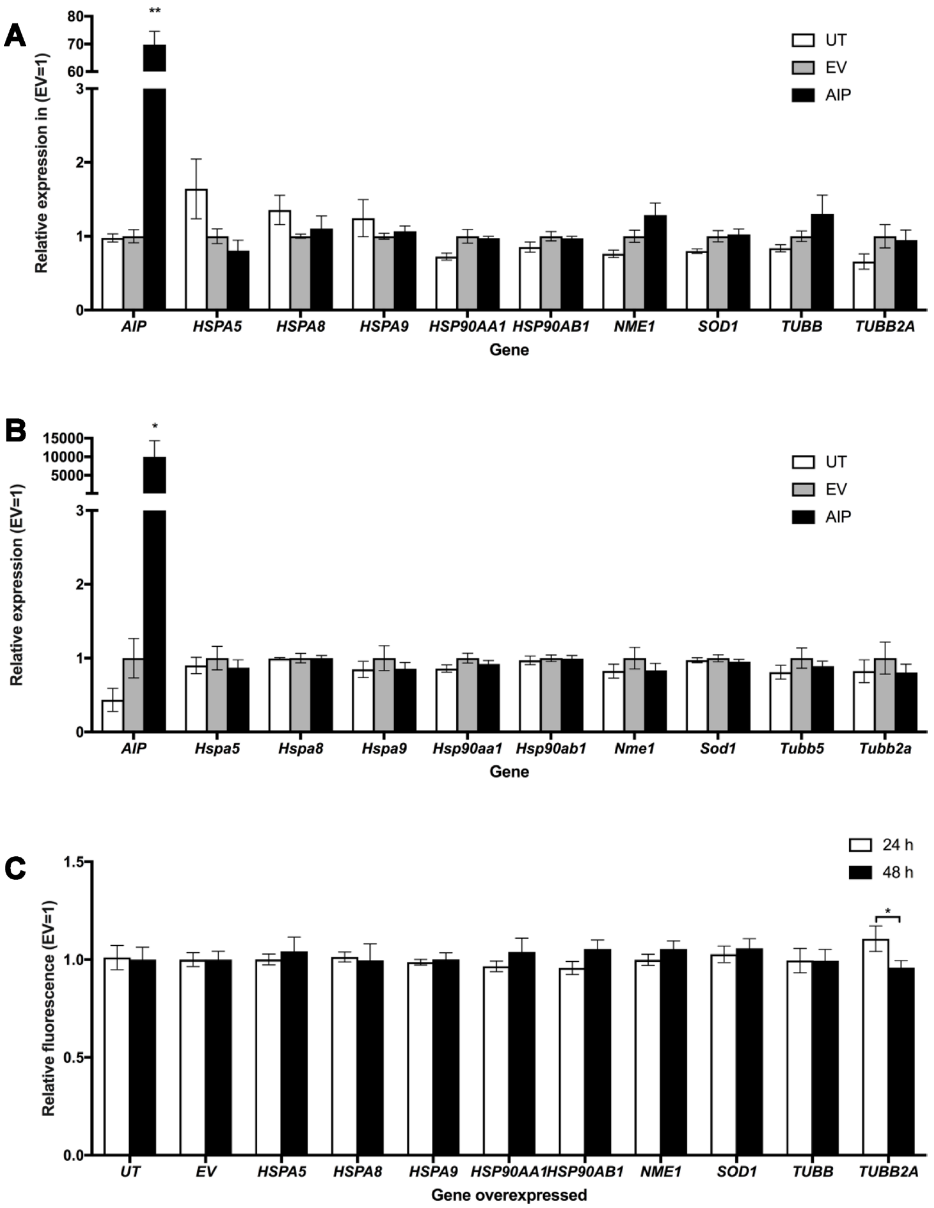

Supplementary Figure 1: Effect of *AIP* overexpression on the expression of its interacting partners in HEK293 (**A**) and GH3 cells (**B**) at the transcriptional level. At 24 h post-transfection, we found no significant differences in the expression of AIP interacting partners ($P > 0.05$ for each gene in overexpressed *AIP* vs. empty vector conditions) with significant *AIP* overexpression in HEK293 and GH3 cells ($P = 0.0088$ and 0.0405 for AIP in overexpressed *AIP* vs. empty vector conditions, for each cell type, respectively). (**C**) Individual overexpression of the AIP interacting partners studied had no significant effect on the proliferation of GH3 cells, compared with empty vector ($P > 0.05$ for each condition vs. empty vector at 24 and 48 h post-transfection). However, overexpression of TUBB2A resulted in reduced cell count at 48 h, compared with the same condition at 24 h ($P = 0.0411$); this effect was not observed for the other genes studied. In graphs: UT: untransfected; EV: empty vector; AIP: *AIP* overexpression.

Supplementary Table 1: Qualitative mass spectrometry results after manual validation. See Supplementary_Table_1

Supplementary Table 2: Comparative intensity values of peptides identified per bait protein. See Supplementary_Table_2

Supplementary Table 3: Transcripts/proteins in expression plasmids. See Supplementary_Table_3
